# SMCis: An Effective Algorithm for Discovery of Cis-Regulatory Modules

**DOI:** 10.1371/journal.pone.0162968

**Published:** 2016-09-16

**Authors:** Haitao Guo, Hongwei Huo, Qiang Yu

**Affiliations:** School of Computer Science and Technology, Xidian University, Xi’an, Shaanxi, China; McGill University, CANADA

## Abstract

The discovery of *cis*-regulatory modules (CRMs) is a challenging problem in computational biology. Limited by the difficulty of using an HMM to model dependent features in transcriptional regulatory sequences (TRSs), the probabilistic modeling methods based on HMMs cannot accurately represent the distance between regulatory elements in TRSs and are cumbersome to model the prevailing dependencies between motifs within CRMs. We propose a probabilistic modeling algorithm called SMCis, which builds a more powerful CRM discovery model based on a hidden semi-Markov model. Our model characterizes the regulatory structure of CRMs and effectively models dependencies between motifs at a higher level of abstraction based on segments rather than nucleotides. Experimental results on three benchmark datasets indicate that our method performs better than the compared algorithms.

## Introduction

**Fig 1:**
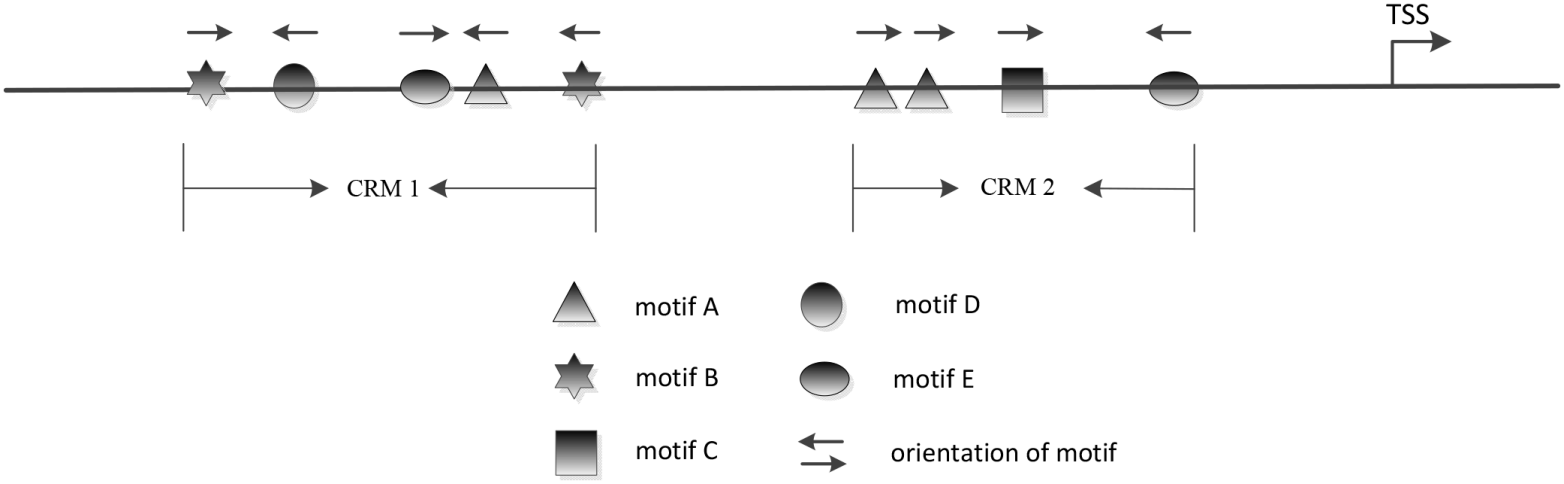
Regulatory structure of a CRM. A CRM is a sequence segment that contains instances of multiple motifs. The orientations of the motifs, the distance between motifs and the relationships between motifs may be key properties of CRMs.

The regulation of gene expression involves the binding of transcription factors (TFs) to transcription factor binding sites (TFBSs) [1, 2]. The TFBSs bound by the same transcription factor usually share a conserved DNA sequence pattern called a DNA motif. In higher eukaryotes, gene expression is cooperatively regulated by a number of transcription factors binding to various TFBSs. These TFBSs are tightly clustered and form *cis*-regulatory modules (CRMs) to recruit bound transcription factors and perform more elaborate and accurate regulation. These CRMs are usually scattered across large genomic regions and have lengths ranging from several tens of base pairs (bp) to several thousands of base pairs. We refer to the functional regions harboring CRMs as transcriptional regulatory sequences (TRSs); TRSs include promoter regions, distal DNA regions such as enhancers located in introns, and even other intergenic regions that are far from transcription start sites (TSSs) but still perform implicit regulatory functions. Playing pivotal roles in the regulation of gene expression, CRMs are believed to have a specific regulatory structure, as shown in Fig 1. The computational discovery of CRMs is a key step for constructing a regulatory network.

Experimental identification of the biochemical features [3] closely associated with CRMs, such as occupancy by transcription factors and histone modifications, is an effective method for the discovery of CRMs. However, the experimental determination of these features is costly and time consuming, and this approach can be limited by the number of antibodies and cell types available. Therefore, it is necessary to discover CRMs with the aid of computational methods.

The computational methods used to predict CRMs face the following challenges. The CRMs have flexible structural organization; in a CRM, partial motifs show order preferences, and the distances between them are not fixed. It is difficult to accurately describe such a CRM structure. Eukaryotic regulatory regions are usually large, and the motifs constituting CRMs are often short and degenerate, typically 4–20 bp long. It is difficult to identify motifs in such a large potential search space [4, 5]. This challenge makes it difficult to look for CRMs by identifying their component motifs directly from sequences.

Most CRM discovery methods take advantage of the following general features of CRMs: i) Clustering of motifs: multiple cooperating transcription factors binding to a CRM may lead to the clustering of motifs in a small sequence region. ii) Evolutionary conservation: functional sequences exhibit a lower frequency of mutations than non-functional sequences over evolutionary time. iii) Available motif profiles (from existing motif databases such as TRANSFAC [6] and JASPAR [7]): it is simpler to search for motif instances by using profile matrices from motif libraries than to perform *de novo* motif prediction using computational methods.

A variety of models and methods have been proposed to predict CRMs [3, 8–15] in eukaryotic genes. Different methods take advantage of different features of CRMs and use diverse search strategies. These methods can be classified into the following three categories according to the search strategies.

One group of methods searches for CRMs based on window clustering and makes use of the clustering of motifs. Some methods, such as MSCAN [16] and MCAST [17], use a simple means of representing a CRM as a region with a high density of motifs within a window. These methods infer CRMs by counting the number of occurrences of given motifs within a sequence window. Other methods use combinatorial search approaches to look for clusters of motifs that co-occur significantly within a given size window; for example, CisMiner [13] detects CRMs by the fuzzy clustering of closely located motifs and CPModule [18] identifies CRMs based on itemset mining. In essence, the methods in this category assume that the motifs within each sequence window are independent and identically distributed. Moreover, it is not a trivial task to determine a reasonable window size and score thresholds.

A second group of methods builds probabilistic models for CRMs and identifies the sequence regions matching a statistical model of a motif cluster better than a background model. Except for a small number of methods based on discriminative models, such as HexDiff [19], regulatory Potential [20] and CRFEM [21], these methods use generative models. The most commonly used generative model is the HMM [22]. The HMM can provide a statistically reliable measure of the occurrences of CRMs and motifs, and it can characterize the regulatory structure of CRMs. Additionally, the expectation-maximization (EM) algorithm used in model learning can automatically estimate a large number of model parameters. The methods based on HMM models often represent TRSs that contains motifs and CRMs as observations generated by a hidden Markov stochastic process. Compared with the window clustering methods, the methods in this category do not require the consideration of window sizes and score thresholds. Early methods, such as CisModule [23] and Cluster-Buster [24], implement simple HMM models with the states describing motifs and intra-module and inter-module backgrounds to infer CRMs. Geometric distributions for state durations in the HMM putatively specify the inter-motif and inter-module distances. However, these methods only model combinations of motifs; they do not consider any preferential ordering of motifs within a CRM. Later methods, such as Stubb [25] and BayCis [26], further extend this model by introducing transitions between motif states.

A third group of methods searches for CRMs in evolutionarily conserved regions. Some methods, such as MorphMS [27] and StubbMS [25] (the multi-species version of Stubb), first identify conserved regions by using pairwise or multiple sequence alignments in the regulatory regions of related genes, then model the motif clusters within those regions by using a TFBS evolution stochastic model to identify conserved CRMs. However, since the regulatory regions of most genes suffer from a large number of events such as shuffling, deletion and duplication, these methods are difficult to align them. To get around this problem, other methods, such as EEL [28] and ReLA [29], have been proposed. These methods align the pre-identified motif instances instead of raw sequences to detect conserved motif cluster regions. Although the methods in this category have shown promising prediction performance for CRMs, they are limited to related species and thus do not always work.

Of all these methods, the probabilistic modeling methods based on HMMs are the most common and most effective. However, the traditional HMM has two drawbacks that limit its prediction performance. First, HMM state durations are implicitly assumed to be geometric distributions. This assumption is unrealistic because the distances between motifs within a CRM may not be well described by a geometric distribution. Second, the HMM is an unwieldy way to model large numbers of dependences. Current methods based on HMMs usually assume that motifs within a CRM are generated independently by corresponding HMMs. However, transcription factors bound to a CRM cooperate with each other to regulate gene expression. This behavior implies that the motifs corresponding to these transcription factors may be correlated. Thus, the independence assumption may cause predictions to be inaccurate. Although the HMM may be extended to model these correlations by adding extra states and parameters, the extended model may require excessive computational work.

To address these problems, this paper presents a probabilistic modeling method called SMCis. The method builds a CRM discovery model based on a hidden semi-Markov model (HSMM) [30]. We use this sophisticated HMM at a higher level of abstraction (i.e., segments rather than nucleotides) to characterize the regulatory structure of CRMs. Unlike general CRM discovery methods, we consider the distances and ordering of motifs within a CRM instead of simply regarding a CRM as a cluster of motifs. Specifically, we infer the CRM structure from the frequencies of motif occurrences and the dependences and distance specificities between motifs within a CRM. The dependences and distance specificities between motifs within a CRM encode gene regulation information. Modeling these features helps to improve the accuracy of CRM discovery. We test our method on three annotated real biological datasets and compare it with current published methods. Experimental results suggest that our method performs better than the compared algorithms.

## Materials and Methods

The HSMM has more modeling power than the HMM and can explicitly model the state durations and the long-range dependencies between observations and states. Thus, the HSMM is a natural and effective approach for modeling TRSs and CRMs. We used an HSMM to characterize the organization of TRSs and the putative transcriptional regulatory structure of CRMs. The structure models the dependencies and distance specificities between motifs within a CRM and describes the internal organization of a CRM.

In this section, we first introduce the details of the model. Then, the algorithms for learning and inference are given. Finally, we describe an algorithm to reduce the search space of the model.

### Construction of the HSMM

The HSMM [30] is an extension of the classical HMM. In contrast to an HMM, each state of which emits one observation, each state of the HSMM can emit strings of observations. In our model, an observation denotes an observed nucleotide. The observations emitted by an HSMM state are governed by a segment model. The segment model gives a joint model for random-length strings of observations. Formally, given an observation sequence *o*_1:*t*_ = *o*_1_*o*_2_…*o*_*t*_ generated by state *s*, the segment model [31] can be expressed as follows:

$$P\left(o_{1:t},t|s\right)=P\left(t|s\right)P\left(o_{1:t}|t,s\right)=d_{s}\left(t\right)e_{s}\left(o_{1:t}\right)$$ (1)

The segment model consists of a duration distribution *d*_*s*_(*t*), describing the probability that observations generated by state *s* have length *t*, and an emission model *e*_*s*_(*o*_1:*t*_), giving the emission probability that state *s* generates the particular observations *o*_1:*t*_.

**Fig 2:**
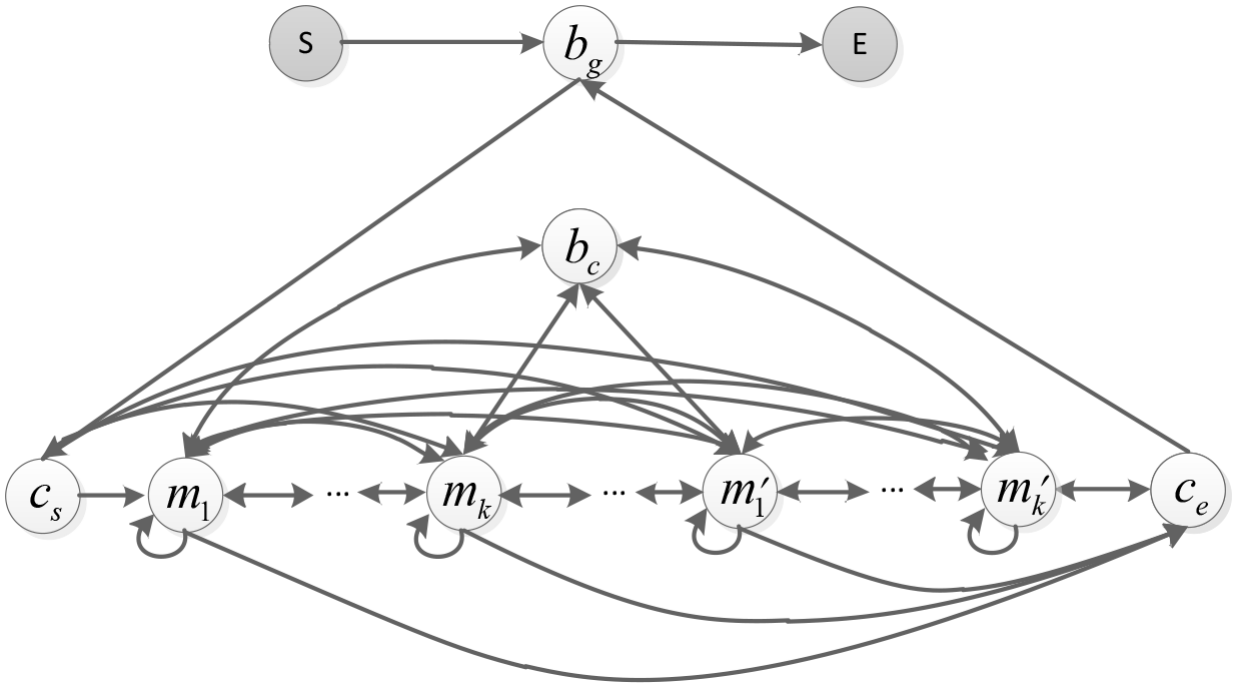
The SMCis HSMM state transition diagram. Nodes represent emission states, including motifs, inter-module backgrounds and intra-module backgrounds. Shadow nodes represent the initial and termination states of the model and the initial and termination states of the CRMs. Arrows indicate permissible transitions between states.

We use an HSMM to describe the regulatory structure of TRSs. Fig 2 shows the HSMM state diagram that describes the regulatory structure of a TRS. In the HSMM, TRSs containing CRMs are organized in a two-level hierarchy. At the top level, each TRS is viewed as a concatenation of CRMs and inter-module (global) backgrounds. At the bottom level, each CRM is considered a combination of motifs and intra-module (local) backgrounds. The motifs and intra-module backgrounds at the bottom level are viewed as nucleotide segments but are not further divided. Formally, in the model, a CRM is denoted by two dummy states, *c*_*s*_ and *c*_*e*_, where *c*_*s*_ initializes a CRM instance and *c*_*e*_ correctly terminates the CRM instance. Let *b*_*g*_ and *b*_*c*_ denote the global and local backgrounds, respectively. We let *M* = {*m*_1_, *m*_2_, …, *m*_*K*_} represent the set of motif states and define *M'* = {*m*_1_*'*, *m*_2_*'*, …, *m*_*K*_*'*} to be its reverse complement. In addition, to make the HSMM well defined, we add the initial state **S** and the termination state **E**. Therefore, the state space of the whole model is denoted by *H* = {**S**, **E**}∪{*c*_*s*_, *c*_*e*_}∪*M* ∪*M'* ∪{*b*_*g*_}∪{*b*_*c*_}.

To capture the dependencies between adjacent motifs within a CRM, we define the direct transitions between motif states, as shown in Fig 2. Dependencies between motifs within a CRM implicitly specify the ordering in the spatial arrangement of these motifs. The ordering of motifs within a CRM may affect combinatorial transcriptional regulation [32]. Thus, modeling the dependencies between motifs helps to uncover the mechanism of TFBS regulation within a CRM.

Motifs within a CRM may comply with specific spacing requirements to allow corresponding transcription factors to bind to them. Several previous studies [33] have suggested that a large number of motif pairs suffer from distance constraints under selection forces and exhibit significant distance specificity in human promoters. To model the motif distance specificity, we approximate the state durations of intra-module backgrounds with a more flexible distribution instead of the geometric distribution implicitly assumed by an HMM.

The HSMM contains three types of emission states: motif state *m*∈*M*∪*M'*, global background state *b*_*g*_ and local background state *b*_*c*_. Once entering an emission state, the HSMM first resorts to a duration model to determine the duration for the visit. Then, it uses a state emission model to emit this number of nucleotides until it transitions to the next state. The specific state duration and state emission models are defined in the following sections.

### State duration model

In the model, state duration models are addressed by the length distributions that represent the length characteristic of nucleotides generated by a particular state.

Position weight matrices (PWMs) [34] of all the motifs are obtained directly from databases (such as TRANSFAC and JASPAR). Given a motif state *m*, let *w*_*m*_ be its length. The following formula defines the probability distribution that a sequence generated by the motif state *m* has length *u*_*m*_:

$$d_{m}\left(u_{m}\right)=\{\begin{array}{cc} 1, & ifu_{m}=w_{m}\\ 0, & ifu_{m}\neq w_{m} \end{array}.$$ (2)

In our model, we do not make any assumption regarding the distance distribution between CRMs and we still use a geometric distribution. The probability that a background sequence generated by the global background state *b*_*g*_ has length *u*_*g*_ is defined as follows:

$$d_{b_{g}}\left(u_{g}\right)={\left(1-p_{g}\right)}^{u-1}p_{g},$$ (3)

where *p*_*g*_ is the parameter of the distribution.

The sequence modeled as an intra-module background defines the distance between adjacent motif instances. The geometric distribution under an HMM is not a good approximation of the distance between motifs. Based on previous research [26], we use a negative binomial distribution to approximate the distribution of the distances between motifs. This distribution is given as follows:

$$d_{b_{c}}\left(u_{c}\right)=\left(\begin{array}{c} u_{c}-1\\ r-1 \end{array}\right)\pi^{u_{c}-r}{\left(1-\pi \right)}^{r},$$ (4)

where *b*_*c*_ is the local background state, *u*_*c*_ is the length of a local background sequence generated by the state *b*_*c*_, and *r* and *π* are the parameters of the distribution function.

### State emission model

The HSMM state emission models define joint probability distributions of nucleotides at particular sites generated by a particular state.

For the global background and local background states, we use the *m*th order and *m'*th order local Markov models, respectively. In a *k*th order local Markov model with *k* = *m* or *m'*, the probability of generating a background sequence *o*_1:*v*_ is given as follows:

$$e_{b}\left(o_{1:v}\right)=\prod_{i=1}^{v}P\left(o_{i}|o_{i-k:i-1}\right),$$ (5)

where *b* denotes the global or local background state.

In Eq (5), *p*(*o*_*i*_|*o*_*i*-*k*:*i*-1_) is the conditional probability of the nucleotide *o*_*i*_ occurring at position *i* given *k* preceding nucleotides *o*_*i*-*k*:*i*-1_. This probability can be computed as follows:

$$P\left(o_{i}|o_{i-k:i-1}\right)=\frac{P\left(o_{i-k:i}\right)}{\sum_{o_{i}=\text{A}}^{\text{T}}P\left(o_{i-k:i-1},o_{i}\right)}.$$ (6)

In Eq (6), *p*(*o*_*i*-*k*:*i*_) and *p*(*o*_*i*-*k*:*i*-*1*_) are estimated from the frequencies of all (*k*+1)-mers and all *k*-mers in an *i*-centered window with length 2*D*, respectively. Here, *D* is a predefined parameter. To optimize the computational efficiency, a sliding window approach is used. The sliding window approach scans the whole sequence in one pass, and the conditional probabilities of nucleotides at all positions are calculated. The detailed steps are as follows:

1. Count each (*k*+1)-mer within the current window, and calculate and store the conditional probability of the nucleotide at each position within the current window.
2. Move the window in a fixed step size, update counts of the (*k*+1)-mers within the current window, and calculate and store the conditional probability of the nucleotide at each position within the current window.
3. Repeat steps i) and ii) until the conditional probabilities of the nucleotides at all positions are calculated.

For the motif state, we use the standard product multinomial (PM) model [35]. The PM model is a simple motif model based on PWMs, which assumes that nucleotides at all positions in the motifs are independent. Given a motif *m*, let its PWM *Θ* = [*θ*_1_, *θ*_2_, …, *θ*_*L*_], where *θ*_*i*_ (1 ≤ *i* ≤ *L*) is a column vector of the frequencies of nucleotides A, T, G and C. The probability of generating a motif instance *o*_1:*L*_ by the motif state *m* is given as follows:

$$e_{m}\left(o_{1:L}\right)=\prod_{i=1}^{L}\theta_{o_{i},i},$$ (7)

where *o*_*i*_ is the nucleotide at position *i* of the motif instance *o*_1:*L*_.

### Inference and learning

In an HMM, model parameters can be estimated by using the Baum-Welch algorithm [36]. The Baum-Welch algorithm uses the EM algorithm [36] to find the maximum likelihood estimate of model parameters. We extend this algorithm to obtain an algorithm called the modified Baum-Welch to estimate the model parameters of our HSMM.

Given a TRS *o*, let *s* denote one of its state paths and let *u* denote the corresponding duration sequence. Let *θ* denote all of the model parameters. The likelihood of a TRS is defined as follows:

$$L\left(\theta \right)=\sum_{s,u}P\left(s,u,o|\theta \right).$$ (8)

Let *θ*^*t*^ represent the value of parameter *θ* at *t*-th iteration, and the corresponding *Q*-function [36] is defined as the likelihood of conditional expectation:

$$Q\left(\theta |\theta^{t}\right)=\sum_{s,u}P\left(s,u|o,\theta^{t}\right)\text{log}P\left(o,s,u|\theta \right).$$ (9)

We compute Eq (9) by using the modified Baum-Welch algorithm based on the EM algorithm to iteratively converge to a locally optimal *θ*.

Once the model is trained, we use the Posterior Viterbi algorithm [37] to discover CRMs in the TRSs. Combining ideas of the Viterbi algorithm [36] and the posterior decoding algorithm [36], the Posterior Viterbi algorithm finds the legal path with the maximum joint posterior probability in the posterior probability space. Formally, given an un-annotated TRS *o*_1:*T*_, the Posterior Viterbi algorithm is used to find a state path *s* = *s*_1:*N*_ and a corresponding state duration sequence *u* = *u*_1:*N*_ according to the following equation:

$$\left(s,u\right)=\underset{N,s=s_{i:N}\in A_{p}}{\text{arg max}}\prod_{i=1}^{N}P\left(s_{i},u_{i}|o_{1:T},\theta \right),$$ (10)

where *N* is the possible number of states and *A*_*p*_ is the set of the allowed posterior paths through the HSMM model.

### Reducing the search space

The HSMM can provide better expressive power than the HMM, but it adds an additional dimension to infer state durations and needs to explicitly evaluate different segmentations in the learning and inferring of the model [31]. Here, each segmentation determines a mapping from a TRS to a set of state labels corresponding to a state path. To reduce the search space, we locate all putative motif instances of given PWMs that are significant matches and may form a CRM before parsing a TRS. In parsing the TRS, the HSMM only considers the state paths through the positions of the motif matches and combines these pre-identified motif instances to identify the best motif clusters as candidate CRMs.

## Results

We tested our method on three real biological datasets: the muscle-specific expression system, the liver-specific expression system and the *Drosophila* early embryonic development system. We refer to these as the muscle dataset, the liver dataset and the *Drosophila* early development dataset, respectively. The sequences in the muscle and liver datasets are from co-regulated genes, and the sequences in the *Drosophila* early development dataset are from orthologous genes.

We chose six methods and compared their prediction performances with that of SMCis on these datasets. These methods encompass a wide spectrum of extant models: BayCis [26], Stubb [25], MSCAN [16], MotEvo [38], Cluster-Buster [24] and ReLA [29].

### Evaluation

To avoid bias toward any particular measure, we used the correlation coefficient (CC) [39] and the F1-score [40] of precision and recall to evaluate the overall prediction performance of our method on all datasets.

Not all of the evaluated methods provide information about motifs within the predicted CRMs; thus, we compared the results at the nucleotide level. Given the prediction results of a method, the CC and F1 scores are defined as follows:

$$\text{CC}=\frac{\text{TP}\times \text{TN-FP}\times \text{FN}}{\sqrt{\left(\text{TP}+\text{FN}\right)\left(\text{FP}+\text{TN}\right)\left(\text{TP}+\text{FP}\right)\left(\text{TN}+\text{FN}\right)}},$$ (11)

$$\text{F}1=\frac{2\times \text{Pr}\times \text{Re}}{\text{Pr}+\text{Re}}.$$ (12)

In Eq (11), TP is the number of nucleotides correctly predicted as CRMs, TN is the number of nucleotides correctly predicted as background, FP is the number of actual background nucleotides mistakenly predicted as CRMs, and FN is the number of actual CRM nucleotides mistakenly predicted as background. In Eq (12), the precision and recall are defined as follows:

- Precision (Pr), Pr = TP / (TP + FP), measures the ratio of correctly predicted CRMs to the total number of predicted CRMs.
- Recall (Re), Re = TP / (TP + FN), measures the ratio of correctly predicted CRMs to the total number of actual CRMs.

Because it uses all four values, TP, TN, FP and FN, the CC is a balanced measure of the overall performance of a method. The CC is interpreted statistically as the correlation between positions in predicted CRMs and positions in actual CRMs. The value of CC ranges from -1 to +1; a value of +1 indicates that the prediction results are fully coincident with the actual results, and a value of -1 means the opposite; the value tends toward 0 when the predictions are close to random.

Precision and recall are antagonistic measures, which means that a higher recall usually comes at the cost of lower precision. The F1-score is defined as the harmonic mean of the antagonistic pairs to balance them. The value of the F1-score ranges from 0 to +1, with +1 indicating perfect predictions.

### Results on the muscle and liver datasets

#### The datasets

The muscle and liver datasets were originally compiled by Wasserman et al. [41, 42], and these datasets have been widely used to evaluate the prediction performance of CRM discovery methods. Klepper et al. [8] expanded the datasets and used them as benchmarks. The two datasets used here were from Klepper et al. [8].

The muscle dataset consists of five motifs and 24 sequences. The five motifs are Mef2, Myf, Sp1, SRF and Tef, which play important roles in the transcriptional regulation of vertebrate muscle gene expression. The average length of the 24 sequences is 850 bp, and these sequences come from rat, human, chicken and cow. The 24 sequences contain 84 instances of the five motifs in total. Each sequence contains one CRM. The average length of these CRMs is 120 bp, ranging from 14 to 294 bp.

The liver dataset includes four motifs and 12 sequences. The four motifs are HNF-1, HNF-3, HNF-4 and C/EBP, which regulate liver-specific gene expression. Each of the 12 sequences, except for the sequence *M*19524 (943 bp long), has a length of 1 Kbp, and the sequences come from human, mouse, rat and chicken. These sequences contain 14 CRMs in total, and each sequence has one or two CRMs. The lengths of these CRMs range from 22 to 176 bp, and the average length is 112 bp.

#### Experimental setup

For the two datasets, we used all sequences as the training and test sets. For the methods Stubb, MSCAN and MotEvo, which depend on a window size, we set the window size to 200 bp and kept the remaining parameters at their default values. The default settings were kept in the other methods.

#### CRM prediction performance

**Fig 3:**
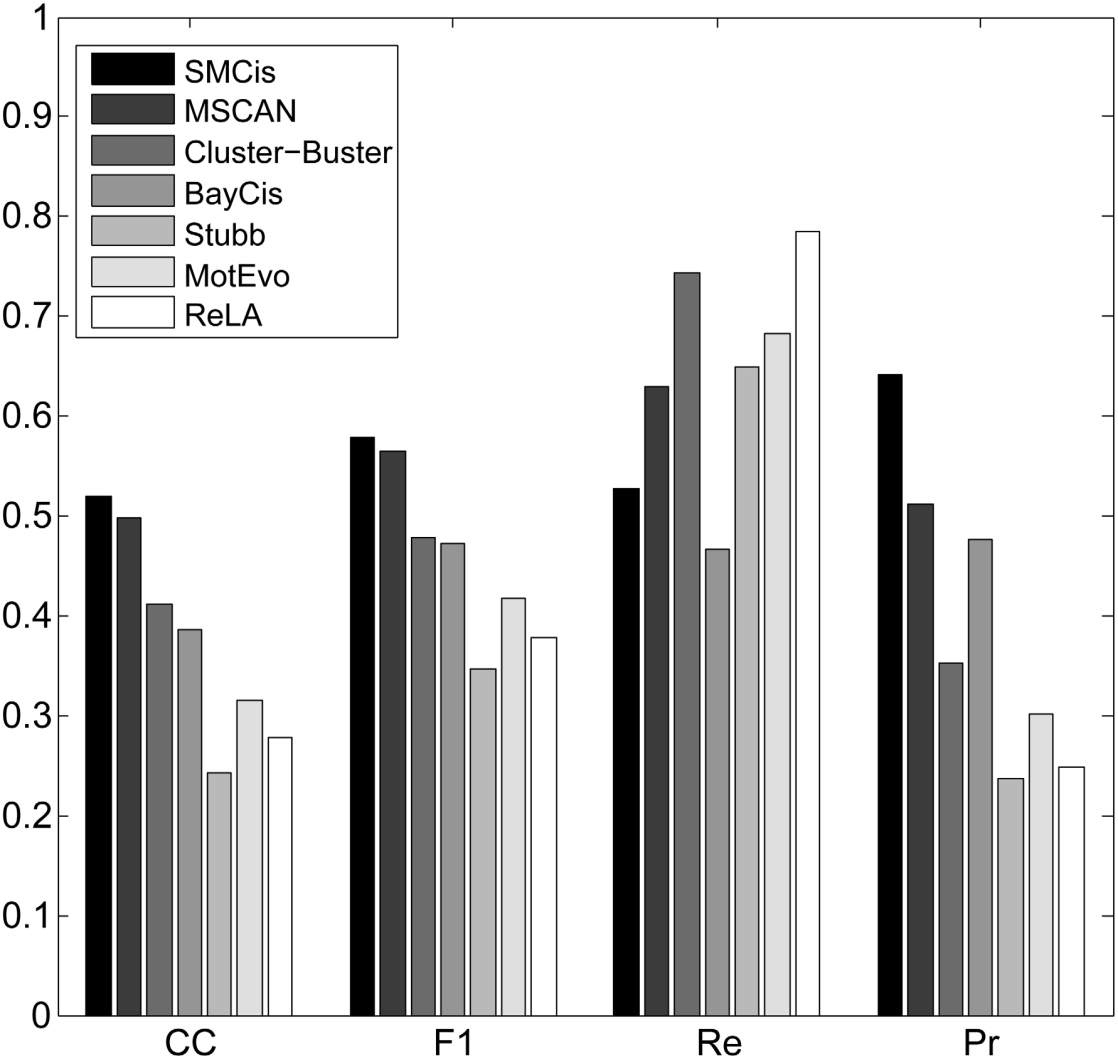
Performance of all methods on the muscle dataset.

**Fig 4:**
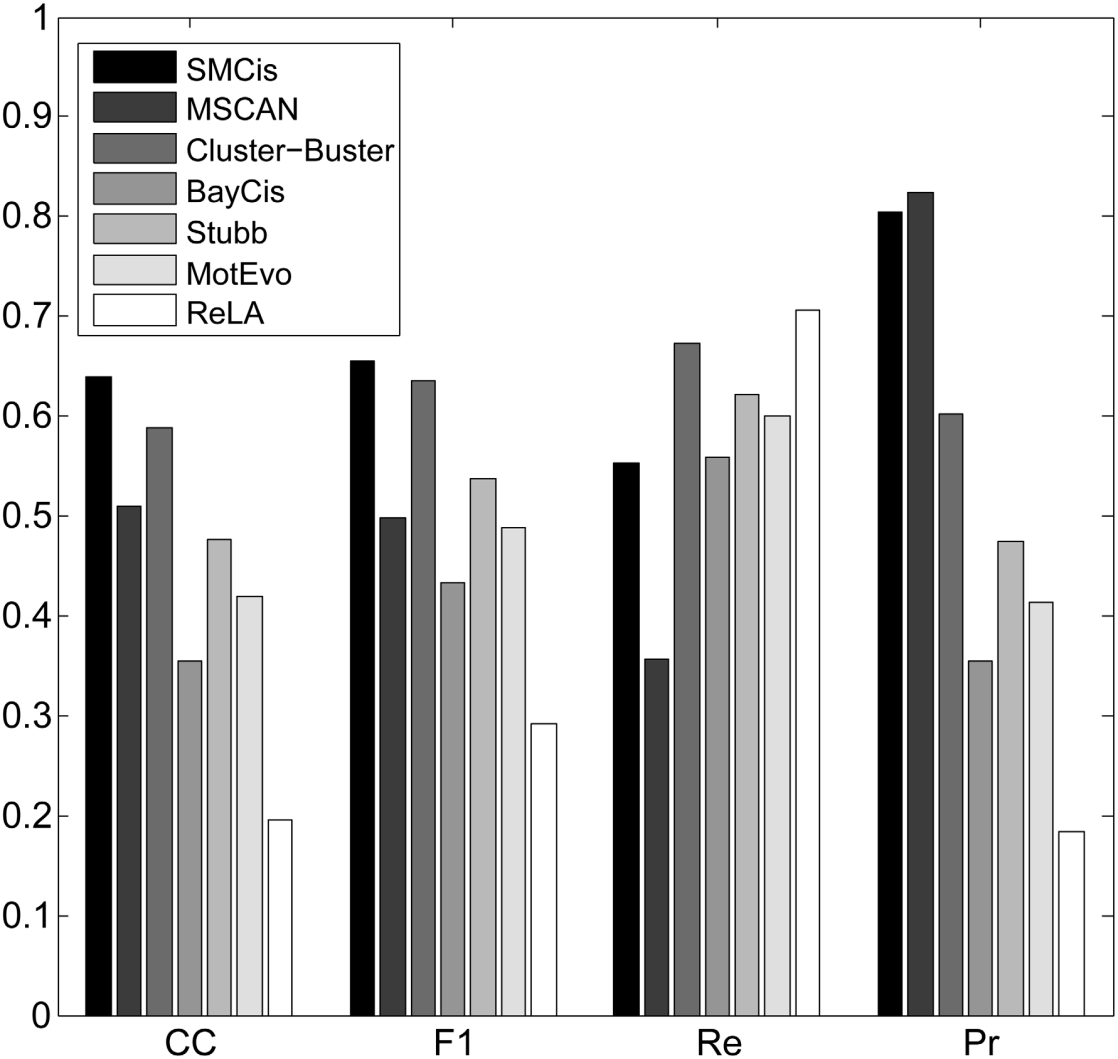
Performance of all methods on the liver dataset.

For all the methods, we calculated the CC, F1-score, precision and recall by summing up all four values TP, TN, FP and FN over all sequences on each of the two datasets, as shown in Figs 3 and 4.

Fig 3 shows that SMCis has the highest CC and F1-score on the muscle dataset; its prediction precision is higher than that of other methods, and its recall is more than half of these methods. As is apparent from Fig 3, different methods have different trends and achieve different balances on this dataset. MotEvo, Stubb, Cluster-Buster and ReLA tend to make predictions with very high recall, but they make a lot of false predictions and do not achieve reasonable balances between precision and recall. BayCis tends to make conservative predictions to ensure high precision, and this is reflected in the fact that its recall is significantly lower than that of the other methods. MSCAN emphasizes neither precision nor recall but provides very high precision while ensuring high recall.

As shown in Fig 4, all methods except ReLA produced better predictions on the liver dataset than the muscle dataset. SMCis had the highest CC score and F1-score on this dataset. Although SMCis had a mid-level recall score, it had high precision that was only slightly lower than that of MSCAN and significantly higher than other methods.

### Results on the *Drosophila* early development dataset

#### The datasets

For the dataset, we used seven motifs—Gt, Hb, Tll, Cad, Kni, Bcd and Kr—which drive the transcriptional regulation of *Drosophila* early embryonic development, and we downloaded PWMs for these motifs from the iDMMPMM database [43]. We selected a subset containing nine genes: *kni*, *Kr*, *hb*, *tll*, *btd*, *eve*, *h*, *ftz* and *prd*, from the genes that orchestrate the anterior-posterior axis patterning in the *Drosophila* early embryo. For each gene, all available orthologous sequences were collected for the learning and inference of the model. We defined each gene search region as a sequence region 40 Kbp long, which was obtained by extracting 20 Kbp downstream and upstream of the TSS. Ortholog information and chromosome coordinates were acquired from the FlyBase database [44]. The seven motifs and nine genes constitute the *Drosophila* early development dataset used in the experiment. We collected CRMs for these genes from the REDfly database [45], merged the overlapping CRMs, and then took them as a benchmark.

#### Experimental setup

SMCis was further compared with the other methods on the *Drosophila* early developmental dataset. For Stubb, we chose its multi-species version StubbMS (a Stubb module, which is referred to as Stubb in the following description). StubbMS extends Stubb by applying phylogenetic comparisons between related organisms. These methods were tested on the dataset as follows:

1. For SMCis and BayCis, on for each gene dataset, we took *D*. *melanogaster* genes as the test set (the REDfly database only collected the annotated CRMs of *D*. *melanogaster* genes and had not yet collected CRMs of other *Drosophila* species genes) and orthologous genes from other species of *Drosophila* as the training set.
2. For MotEvo and Cluster-Buster, we tested them on the whole orthologous group but only focused on the predictions on *D*. *melanogaster* genes.
3. For Stubb, which requires a pairwise sequence alignment, we selected the species used in the original paper (*D*. *melanogaster* and *D*. *virilis*) and tested Stubb on the corresponding genes.
4. For MSCAN, which predicts CRMs on a single sequence, we tested it only on the corresponding *D*. *melanogaster* genes.
5. For ReLA, on each gene dataset, we designated the corresponding *D*. *melanogaster* gene as the reference sequence, and the other *Drosophila* species genes were compared with the reference sequence.

For the methods that depend on a window size, we set the window size to 800 bp (approximately the average length of CRMs on this dataset) and kept the remaining parameters at their default values. The default settings were maintained for the other methods.

#### CRM prediction performance

For each method, we counted TP, FP, TN and FN on each gene dataset and then calculated the CC scores of the predicted results on the gene dataset. To evaluate the overall performance of all the methods on the whole dataset, we also calculated all the measures for each method on the whole *Drosophila* early development dataset.

**Fig 5:**
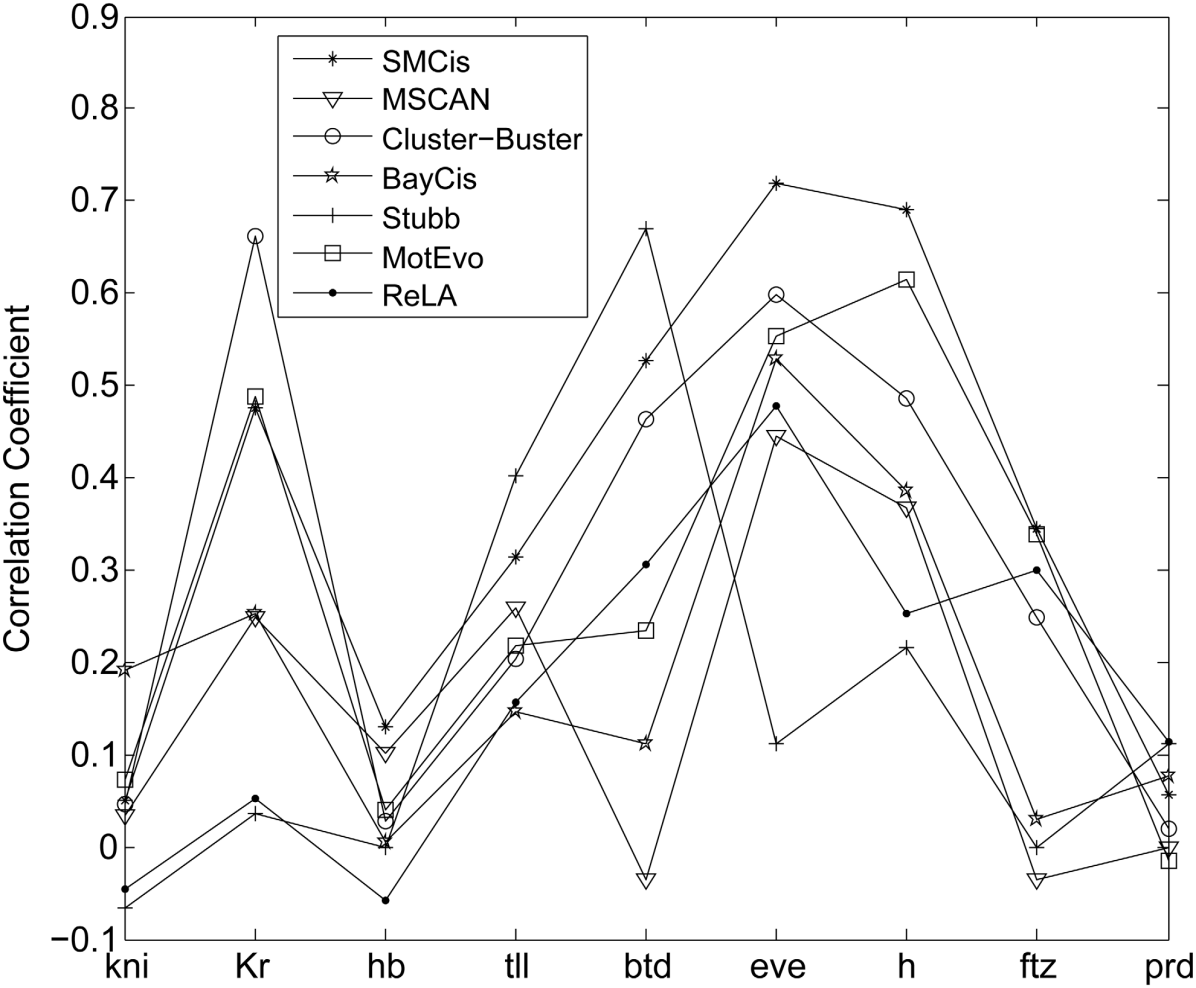
CC scores of all methods, calculated for single genes on the *Drosophila* early development dataset.

Fig 5 shows CC scores for all the methods on each gene dataset. Overall, SMCis achieved a better prediction performance than the other methods. SMCis had the highest CC scores on four of the nine genes tested: *hb*, *eve*, *h* and *ftz*. It had the second best CC scores on *btd* and *tll* and the third best on *kni* and *Kr*.

Fig 5 shows that the prediction performance of all methods exhibited a relatively consistent tendency on most gene datasets. We speculate that the properties of the CRMs of different genes, such as CRM length and the number of motifs involved, may have a significant impact on the performance of CRM discovery methods. Generally, the CRMs that are long and contain multiple instances of known motifs are easier to predict. For instance, the *eve* enhancer *eve*_*stripe*_3+7 contains up to 49 instances of the four motifs, including *kni* and *hb*, and all the methods correctly predicted the CRM. Although it is difficult to design a perfect search strategy that is insensitive to data, most of the probabilistic modeling methods exhibited good adaptability.

**Fig 6:**
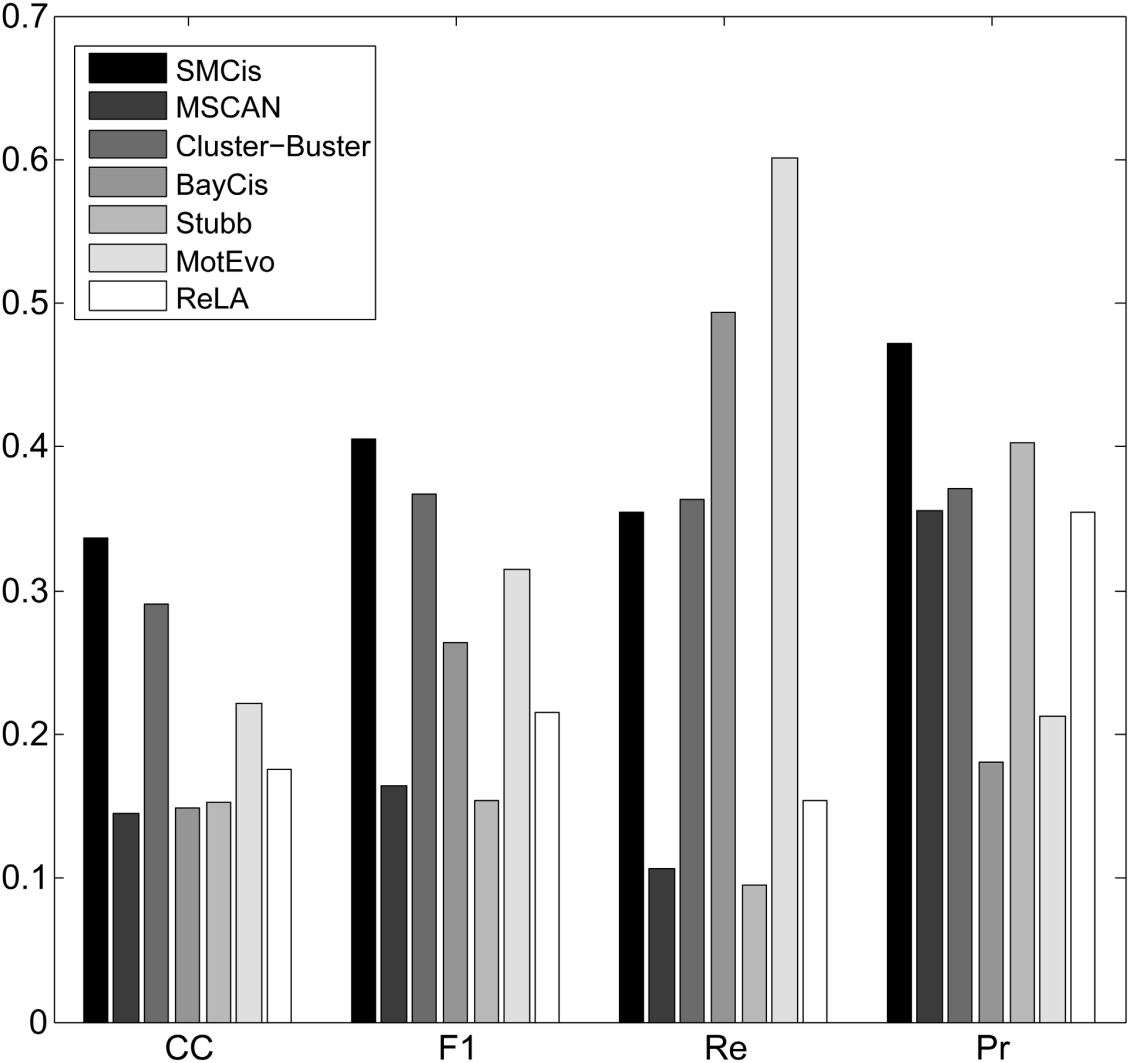
Performance of all methods on the whole *Drosophila* early development dataset.

Fig 6 shows all of the measures for these methods on the whole dataset. The CC scores and F1-scores of all the methods dropped more sharply on this dataset compared with the results on the muscle and liver datasets. Despite this, the scores were higher than all other compared methods for both CC and F1-score. SMCis still achieved stable prediction performance; it had the highest prediction precision while ensuring a reasonable recall.

Compared with the muscle and liver datasets, the sequences on the *Drosophila* early development dataset are much longer and the CRM lengths are more varied, which may affect the prediction performances of the methods. On this dataset, the methods showed significant differences in prediction performance. ReLA achieved better performance than most of the methods, despite its poor performance on the muscle and liver datasets. In contrast to its good performance on the muscle dataset, MSCAN performed very poorly on this dataset; its CC score was the lowest, and its F1-score was only slightly higher than that of Stubb, which had the lowest CC score. One explanation for this may be that it is difficult for window-based clustering methods to determine reasonable window sizes and score thresholds on this dataset. MotEvo also uses a window-based clustering strategy, but it achieved better performance than MSCAN. MotEvo builds a Bayesian probability model to score motifs clustering within a sliding window, which also illustrates that probabilistic modeling methods may have better adaptability to different types of data. Taking advantage of the evolutionary conservation between species, Stubb had high prediction precision (only second to SMCis), but its recall score was the lowest out of all the methods. SMCis does not directly make use of conservation by aligning sequences, but it implicitly considers the evolutionary conservation between species by characterizing the conserved regulatory structures of co-regulated or orthologous sequences based on the HSMM. This additional information helps to improve the prediction performance of SMCis.

## Discussion and Conclusions

In this paper, we present SMCis, a probabilistic modeling method for predicting CRMs that builds a more powerful CRM discovery model based on an HSMM. In this model, we characterize the regulatory structure of a TRS at a higher level of abstraction (sequence segments rather than nucleotides). Our model views a TRS as a combination of CRMs and inter-module backgrounds and further represents a CRM as a combination of motifs and intra-module backgrounds. Paying more attention to the modeling of the CRM internal structure, we consider not only dependencies between motifs within a CRM but also the distance specificities between the motifs. Compared with other probabilistic modeling methods for CRM discovery, SMCis has the following advantages.

1. The level of abstraction at sequence segments rather than single nucleotides makes the model representations more natural. Representing the regulatory structure of a TRS with a hierarchical organization and explicitly defining CRM states makes the overall architecture of the model clearer.
2. Compared with other methods based on HMMs, our model is more flexible. In the model, we can build an individual model for each type of segment (corresponding to the states of the HSMM model). For example, we can introduce more sophisticated models to capture dependencies between nucleotide sites within a motif. Moreover, our model can use any duration distribution with a specific meaning or a combination of distributions; it is not limited to the implicit geometric distribution in the HMM.

To further improve the prediction performance of CRMs, we will continue working on the following issues in follow-up studies. We will collect more CRM annotations and use more systematic approaches, such as k-fold cross validation, to aid in the selection of model parameters. We will also consider using a Bayesian approach to add *a priori* information and other soft constraints, which will make our method more adaptable to new data because it more easily leads to over-fitting to the training data completely based on the likelihood of the observed data.

## Supporting Information

S1 Datasets The real biological datasets used in our experiments, including the liver and muscle datasets [8] and the *Drosophila* early development dataset from the database REDFly [45]. (ZIP)

S1 File The test results of all methods. (ZIP)

S1 Program The executable program of SMCis. (ZIP)

S1 Text Supplement for SMCis and the experiment. (DOC)
