# Supplementary material for: SMCis: An Effective Algorithm for Discovery of Cis-Regulatory Modules
Source: PLoS One. 2016 Sep 16;11(9):e0162968. doi: 10.1371/journal.pone.0162968 (PMC5026350; doi:10.1371/journal.pone.0162968)
Supplement: S1 Text — (DOC) [file pone.0162968.s004.doc]

## Constant parameters of the model

The model has the following constant parameters:

1. The probability of initializing a CRM *p_i_*. The length of inter-module background sequences is geometrically distributed with the parameter *p_g_*, so *p_i_* = 1 - *p_g_* and 1/*p_i_* is the mean of background length.
2. The probability of terminating a CRM *p_t_*. *p_t_* potentially specifies the mean number of motif instances within a CRM.

The values of these parameters were set based on empirical guesses. In the model their default values were set as follows: *p_i_* = 0.001, *p_t_* = 0.2, which generally seemed to produce good results.

In addition, in our model the intra-module background is modeled as a negative binomial distribution with parameters *n* and *p*. The distribution is fitted to the actual spacer length data of D. melanogaster genes. The final *n* and *p* values were set to 2 and 0.05, respectively.

## Time performance of the algorithm

All methods were tested on a machine with Intel Xeon E5640 processor, 16G memory and Ubuntu 14.04 64 bit OS, and their running time on the muscle, liver and Drosophila datasets is shown in Table 1.

**Table 1. The running time of all methods on the muscle, liver and Drosophila datasets.**

|  | **SMCis** | **MSCAN** | **Cluster-Buster** | **BayCis** | **Stubb** | **MotEvo** | **ReLA** |
| --- | --- | --- | --- | --- | --- | --- | --- |
| **muscle** | 32.38s | 0.48s | 0.06s | 8.49m | 0.88s | 11.01s | 23.08s |
| **liver** | 3.67s | 0.43s | 0.03s | 7.32m | 0.57s | 6.35s | 13.26s |
| **Drosophila** | 5.29m | 0.60s | 0.14s | 6.97h | 35.85s | 30.29m | 4.27m |

Time units, s: seconds; m: minutes; h: hours.

Moreover, in order to test the running time of all methods varying with the number of sequences, we constructed 10 synthetic datasets in which the numbers of sequences gradually increase. Each dataset consists of a set of sequences (the number of sequences from 10 to 100 in 10 increments) and a motif set with 10 PWMs which were extracted from the TRANSFAC database. Each sequence has a fixed length of 40kbp and contains 0-3 CRMs. The CRM lengths range is from 200 bp to 1500 bp. Each CRM has about 15 instances of 2-6 different motifs and the average distance between these motif instances is about 50 bp. Furthermore, in order to simulate some motif co-occurring as motif pair, predefined motif pairs making up about 30% of the motif instances were implanted into each CRM. Inter-module and intra-module background sequences are modeled as 3rd order Markov models and their model parameters are estimated from D. melanogaster intergenic regions. The results of the experiment are shown in Table 2.

**Table 2.The running time of all methods on 10 synthetic datasets.**

| **# of seq** | **SMCis** | **MSCAN** | **Cluster-Buster** | **BayCis** | **Stubb** | **MotEvo** | **ReLA** |
| --- | --- | --- | --- | --- | --- | --- | --- |
| 10 | 6.98m | 1.09s | 2.57s | 6.04h | 2.18m | 25.42m | 3.79m |
| 20 | 13.02m | 1.89s | 4.22s | > | 4.35m | 46.24m | 6.44m |
| 30 | 18.63m | 1.94s | 7.35s | > | 6.38m | 1.14h | - |
| 40 | 27.28m | 2.61s | 11.71s | > | 8.62m | 1.56h | - |
| 50 | 34.49m | 2.82s | 11.83s | > | 10.78m | 1.91h | - |
| 60 | 47.93m | 3.34s | 16.19s | > | 12.79m | 2.35h | - |
| 70 | 55.22m | 3.86s | 17.88s | > | 14.89m | 2.77h | - |
| 80 | 64.36m | 4.51s | 17.68s | > | 17.69m | 3.06h | - |
| 90 | 73.61m | 4.55s | 23.34s | > | 19.35m | 3.43h | - |
| 100 | 82.03m | 4.95s | 22.32s | > | 21.22m | 3.82h | - |

Time units, s: seconds; m: minutes; h: hours. Note, running time of BayCis is above 12 hours when the number of sequence is greater than 20, denoted as ‘>’ for these datasets. For ReLA, only a maximum of 25 sequences between 200 to 50000 bp long are accepted, denoted as ‘-’ for these datasets with more than 20 sequences.
