# Supplementary material for: SMCis: An Effective Algorithm for Discovery of Cis-Regulatory Modules
Source: PLoS One. 2016 Sep 16;11(9):e0162968. doi: 10.1371/journal.pone.0162968 (PMC5026350; doi:10.1371/journal.pone.0162968)
Supplement: S1 File — (ZIP) [file pone.0162968.s002.zip › S1_File/stubb/drosophila/output/dmel_btd_peaks.html]

```
# Min_number_factors 3
# Free_energy_cutoff 10 Factor_min_occ 0.5
# Window_shift 100 Window_size 500
>
*********************** Rank 1 [Score 17.9006] PostScript Profile *******************
>X:9568219,9608218	Position: 16300	Nucl: 783	Word_av_length: 0.00	Free Energy: 17.9006
bcd            	0.0082	5.2364
Kr             	0.0010	0.8778
tll            	0.0057	2.8556
Background     	0.9851	709.1078
>XL_group3a:794548,834547	Position: 15302	Nucl: 93	
tll            	0.0057	1.6866
Background     	0.9851	75.0009
>XL_group3a:794548,834547	Position: 15413	Nucl: 49	
bcd            	0.0082	0.8177
Background     	0.9851	42.7335
>XL_group3a:794548,834547	Position: 15485	Nucl: 47	
bcd            	0.0082	0.5040
Background     	0.9851	43.1245
>XL_group3a:794548,834547	Position: 15618	Nucl: 94	
bcd            	0.0082	1.0493
Background     	0.9851	81.4595
>XL_group3a:794548,834547	Position: 15748	Nucl: 85	
bcd            	0.0082	0.9949
tll            	0.0057	1.0417
Background     	0.9851	67.7511
>XL_group3a:794548,834547	Position: 15901	Nucl: 54	
Background     	0.9851	53.6473
<
>
*********************** Rank 2 [Score 14.3031] PostScript Profile *******************
>X:9568219,9608218	Position: 1400	Nucl: 786	Word_av_length: 0.00	Free Energy: 14.3031
cad            	0.0125	7.5261
kni            	0.0080	4.7978
gt             	0.0032	1.9376
tll            	0.0082	4.9128
Background     	0.9673	581.4584
<
```
