# Supplementary material for: SMCis: An Effective Algorithm for Discovery of Cis-Regulatory Modules
Source: PLoS One. 2016 Sep 16;11(9):e0162968. doi: 10.1371/journal.pone.0162968 (PMC5026350; doi:10.1371/journal.pone.0162968)
Supplement: S1 File — (ZIP) [file pone.0162968.s002.zip › S1_File/stubb/drosophila/output/dmel_eve_peaks.html]

```
# Min_number_factors 3
# Free_energy_cutoff 10 Factor_min_occ 0.5
# Window_shift 100 Window_size 500
>
*********************** Rank 1 [Score 46.6255] PostScript Profile *******************
>2R:5846824,5886823	Position: 16800	Nucl: 786	Word_av_length: 0.00	Free Energy: 46.6255
cad            	0.0065	3.9953
kni            	0.0133	6.1477
hb             	0.0022	0.9956
Background     	0.9780	662.6981
>3:10887764,10927763	Position: 15210	Nucl: 699	
cad            	0.0065	4.2786
kni            	0.0133	10.4849
hb             	0.0022	1.7848
Background     	0.9780	508.4386
>3:10887764,10927763	Position: 15931	Nucl: 78	
Background     	0.9780	74.9550
<
>
*********************** Rank 2 [Score 34.4265] PostScript Profile *******************
>2R:5846824,5886823	Position: 16200	Nucl: 781	Word_av_length: 0.00	Free Energy: 34.4265
kni            	0.0114	7.2416
hb             	0.0037	2.2236
Background     	0.9828	664.3359
>3:10887764,10927763	Position: 15023	Nucl: 105	
cad            	0.0021	0.7356
kni            	0.0114	1.0259
Background     	0.9828	80.8050
>3:10887764,10927763	Position: 15189	Nucl: 44	
Background     	0.9828	42.9747
>3:10887764,10927763	Position: 15433	Nucl: 16	
Background     	0.9828	15.8997
>3:10887764,10927763	Position: 15487	Nucl: 13	
Background     	0.9828	12.6681
>3:10887764,10927763	Position: 15542	Nucl: 292	
cad            	0.0021	1.1339
kni            	0.0114	3.5443
hb             	0.0037	1.1650
Background     	0.9828	225.7060
<
>
*********************** Rank 3 [Score 21.7296] PostScript Profile *******************
>2R:5846824,5886823	Position: 30700	Nucl: 787	Word_av_length: 0.00	Free Energy: 21.7296
cad            	0.0198	12.1388
hb             	0.0146	8.9721
Background     	0.9648	592.1454
<
```
