# Supplementary material for: SMCis: An Effective Algorithm for Discovery of Cis-Regulatory Modules
Source: PLoS One. 2016 Sep 16;11(9):e0162968. doi: 10.1371/journal.pone.0162968 (PMC5026350; doi:10.1371/journal.pone.0162968)
Supplement: S1 File — (ZIP) [file pone.0162968.s002.zip › S1_File/stubb/drosophila/output/dmel_gt_peaks.html]

```
# Min_number_factors 3
# Free_energy_cutoff 10 Factor_min_occ 0.5
# Window_shift 100 Window_size 500
>
*********************** Rank 1 [Score 10.3387] PostScript Profile *******************
>X:2301146,2341145	Position: 35100	Nucl: 787	Word_av_length: 0.00	Free Energy: 10.3387
kni            	0.0051	3.4852
Kr             	0.0094	6.3642
tll            	0.0016	1.1179
Background     	0.9838	667.3115
<
```
