# Supplementary material for: SMCis: An Effective Algorithm for Discovery of Cis-Regulatory Modules
Source: PLoS One. 2016 Sep 16;11(9):e0162968. doi: 10.1371/journal.pone.0162968 (PMC5026350; doi:10.1371/journal.pone.0162968)
Supplement: S1 File — (ZIP) [file pone.0162968.s002.zip › S1_File/stubb/drosophila/output/dmel_h_peaks.html]

```
# Min_number_factors 3
# Free_energy_cutoff 10 Factor_min_occ 0.5
# Window_shift 100 Window_size 500
>
*********************** Rank 1 [Score 15.7012] PostScript Profile *******************
>3L:8648859,8688858	Position: 15700	Nucl: 781	Word_av_length: 0.00	Free Energy: 15.7012
bcd            	0.0015	0.6825
Kr             	0.0069	2.9844
tll            	0.0046	2.3497
Background     	0.9870	723.2124
>XR_group8:8891229,8931228	Position: 13557	Nucl: 76	
Kr             	0.0069	0.7334
Background     	0.9870	68.6730
>XR_group8:8891229,8931228	Position: 13678	Nucl: 53	
Kr             	0.0069	0.9562
Background     	0.9870	43.4335
>XR_group8:8891229,8931228	Position: 13818	Nucl: 54	
Kr             	0.0069	0.9579
Background     	0.9870	44.1098
>XR_group8:8891229,8931228	Position: 13932	Nucl: 154	
Kr             	0.0069	1.8016
tll            	0.0046	1.6172
Background     	0.9870	119.9416
>XR_group8:8891229,8931228	Position: 14099	Nucl: 38	
tll            	0.0046	1.0062
Background     	0.9870	28.1337
>XR_group8:8891229,8931228	Position: 14179	Nucl: 29	
bcd            	0.0015	0.8358
Background     	0.9870	21.6408
>XR_group8:8891229,8931228	Position: 14223	Nucl: 40	
Background     	0.9870	39.5902
<
>
*********************** Rank 2 [Score 13.7161] PostScript Profile *******************
>3L:8648859,8688858	Position: 39700	Nucl: 787	Word_av_length: 0.00	Free Energy: 13.7161
cad            	0.0015	1.5144
hb             	0.0090	3.4662
gt             	0.0012	1.5186
Background     	0.9875	723.9792
>XR_group8:8891229,8931228	Position: 39830	Nucl: 145	
hb             	0.0090	0.8266
Kr             	0.0007	0.8087
Background     	0.9875	126.3944
>XR_group8:8891229,8931228	Position: 39988	Nucl: 631	
hb             	0.0090	8.5278
Background     	0.9875	549.7731
<
>
*********************** Rank 3 [Score 10.4045] PostScript Profile *******************
>3L:8648859,8688858	Position: 11100	Nucl: 779	Word_av_length: 0.00	Free Energy: 10.4045
cad            	0.0053	2.8752
hb             	0.0032	1.8328
Kr             	0.0019	1.8454
tll            	0.0050	1.8827
Background     	0.9846	700.4972
>XR_group8:8891229,8931228	Position: 8723	Nucl: 19	
Background     	0.9846	17.5870
>XR_group8:8891229,8931228	Position: 8789	Nucl: 40	
Background     	0.9846	36.6150
>XR_group8:8891229,8931228	Position: 8912	Nucl: 37	
Background     	0.9846	36.8370
>XR_group8:8891229,8931228	Position: 8982	Nucl: 54	
cad            	0.0053	0.8732
Background     	0.9846	46.1990
>XR_group8:8891229,8931228	Position: 9129	Nucl: 18	
Background     	0.9846	15.4062
>XR_group8:8891229,8931228	Position: 9157	Nucl: 50	
Background     	0.9846	44.9816
>XR_group8:8891229,8931228	Position: 9221	Nucl: 72	
hb             	0.0032	0.8511
tll            	0.0050	0.8591
Background     	0.9846	54.3222
>XR_group8:8891229,8931228	Position: 9305	Nucl: 227	
cad            	0.0053	1.4858
tll            	0.0050	2.8312
Background     	0.9846	183.1681
<
```
