# Supplementary material for: SMCis: An Effective Algorithm for Discovery of Cis-Regulatory Modules
Source: PLoS One. 2016 Sep 16;11(9):e0162968. doi: 10.1371/journal.pone.0162968 (PMC5026350; doi:10.1371/journal.pone.0162968)
Supplement: S1 File — (ZIP) [file pone.0162968.s002.zip › S1_File/stubb/drosophila/output/dmel_kni_peaks.html]

```
# Min_number_factors 3
# Free_energy_cutoff 10 Factor_min_occ 0.5
# Window_shift 100 Window_size 500
>
*********************** Rank 1 [Score 26.4426] PostScript Profile *******************
>3L:20665430,20705429	Position: 15300	Nucl: 784	Word_av_length: 0.00	Free Energy: 26.4426
cad            	0.0013	0.6555
bcd            	0.0008	0.9117
hb             	0.0059	2.7635
Kr             	0.0057	2.9931
tll            	0.0048	3.0083
Background     	0.9816	688.0820
>XR_group8:8078976,8118975	Position: 15502	Nucl: 18	
hb             	0.0059	0.7497
Background     	0.9816	11.0863
>XR_group8:8078976,8118975	Position: 15532	Nucl: 28	
Background     	0.9816	25.9115
>XR_group8:8078976,8118975	Position: 15585	Nucl: 99	
hb             	0.0059	1.3241
Kr             	0.0057	0.6578
Background     	0.9816	78.2345
>XR_group8:8078976,8118975	Position: 15703	Nucl: 102	
hb             	0.0059	0.9590
tll            	0.0048	0.5128
Background     	0.9816	87.0324
>XR_group8:8078976,8118975	Position: 15826	Nucl: 29	
hb             	0.0059	0.5716
Background     	0.9816	20.8099
>XR_group8:8078976,8118975	Position: 15885	Nucl: 57	
Background     	0.9816	55.8435
>XR_group8:8078976,8118975	Position: 16011	Nucl: 88	
tll            	0.0048	0.8194
Background     	0.9816	78.6019
>XR_group8:8078976,8118975	Position: 16158	Nucl: 158	
Kr             	0.0057	2.4219
tll            	0.0048	1.0554
Background     	0.9816	116.8085
<
>
*********************** Rank 2 [Score 19.6936] PostScript Profile *******************
>3L:20665430,20705429	Position: 15800	Nucl: 785	Word_av_length: 0.00	Free Energy: 19.6936
cad            	0.0124	8.0111
Kr             	0.0053	2.6030
tll            	0.0023	1.5096
Background     	0.9801	672.4687
>XR_group8:8078976,8118975	Position: 16066	Nucl: 34	
Background     	0.9801	26.2311
>XR_group8:8078976,8118975	Position: 16158	Nucl: 122	
cad            	0.0124	1.5913
Kr             	0.0053	1.9433
Background     	0.9801	88.3716
>XR_group8:8078976,8118975	Position: 16341	Nucl: 20	
Background     	0.9801	17.2401
>XR_group8:8078976,8118975	Position: 16389	Nucl: 90	
cad            	0.0124	0.8948
Background     	0.9801	80.9704
<
```
