# Supplementary material for: SMCis: An Effective Algorithm for Discovery of Cis-Regulatory Modules
Source: PLoS One. 2016 Sep 16;11(9):e0162968. doi: 10.1371/journal.pone.0162968 (PMC5026350; doi:10.1371/journal.pone.0162968)
Supplement: S1 File — (ZIP) [file pone.0162968.s002.zip › S1_File/stubb/drosophila/output/dmel_prd_peaks.html]

```
# Min_number_factors 3
# Free_energy_cutoff 10 Factor_min_occ 0.5
# Window_shift 100 Window_size 500
>
*********************** Rank 1 [Score 15.7986] PostScript Profile *******************
>2L:12062995..12102994	Position: 4500	Nucl: 786	Word_av_length: 0.00	Free Energy: 15.7986
cad            	0.0087	4.3294
hb             	0.0007	0.5308
Background     	0.9901	736.2335
>4_group2:850563..890562	Position: 3371	Nucl: 627	
cad            	0.0087	5.5637
hb             	0.0007	0.6424
Background     	0.9901	567.3643
>4_group2:850563..890562	Position: 4023	Nucl: 677	
cad            	0.0087	5.8307
Background     	0.9901	620.2748
>4_group2:850563..890562	Position: 4724	Nucl: 563	
cad            	0.0087	5.6643
Background     	0.9901	507.8666
<
>
*********************** Rank 2 [Score 14.7310] PostScript Profile *******************
>2L:12062995..12102994	Position: 21700	Nucl: 786	Word_av_length: 0.00	Free Energy: 14.7310
bcd            	0.0062	4.1194
Kr             	0.0058	3.5383
tll            	0.0018	1.5500
Background     	0.9863	707.4352
>4_group2:850563..890562	Position: 25370	Nucl: 33	
Background     	0.9863	32.8069
>4_group2:850563..890562	Position: 25434	Nucl: 106	
bcd            	0.0062	0.5754
Kr             	0.0058	0.5202
Background     	0.9863	98.4773
>4_group2:850563..890562	Position: 25617	Nucl: 431	
bcd            	0.0062	2.9451
Kr             	0.0058	2.9886
tll            	0.0018	0.5944
Background     	0.9863	374.7467
<
```
