# Supplementary material for: SMCis: An Effective Algorithm for Discovery of Cis-Regulatory Modules
Source: PLoS One. 2016 Sep 16;11(9):e0162968. doi: 10.1371/journal.pone.0162968 (PMC5026350; doi:10.1371/journal.pone.0162968)
Supplement: S1 File — (ZIP) [file pone.0162968.s002.zip › S1_File/stubb/drosophila/output/dmel_tll_peaks.html]

```
# Min_number_factors 3
# Free_energy_cutoff 10 Factor_min_occ 0.5
# Window_shift 100 Window_size 500
>
*********************** Rank 1 [Score 12.1599] PostScript Profile *******************
>3R:26658037,26698036	Position: 17300	Nucl: 782	Word_av_length: 0.00	Free Energy: 12.1599
bcd            	0.0043	2.8910
Kr             	0.0017	1.0516
Background     	0.9939	751.1954
>2:479330,519329	Position: 14809	Nucl: 23	
Background     	0.9939	22.7219
>2:479330,519329	Position: 14895	Nucl: 20	
Background     	0.9939	19.8591
>2:479330,519329	Position: 14941	Nucl: 26	
Background     	0.9939	25.8224
>2:479330,519329	Position: 15038	Nucl: 16	
Background     	0.9939	15.9189
>2:479330,519329	Position: 15085	Nucl: 77	
Kr             	0.0017	0.9749
Background     	0.9939	67.1103
>2:479330,519329	Position: 15195	Nucl: 19	
Background     	0.9939	17.4889
>2:479330,519329	Position: 15240	Nucl: 37	
Background     	0.9939	36.7587
>2:479330,519329	Position: 15314	Nucl: 39	
Background     	0.9939	38.8588
>2:479330,519329	Position: 15402	Nucl: 42	
Background     	0.9939	41.8022
>2:479330,519329	Position: 15492	Nucl: 225	
bcd            	0.0043	2.1111
Background     	0.9939	210.0365
<
```
