# Supplementary material for: SMCis: An Effective Algorithm for Discovery of Cis-Regulatory Modules
Source: PLoS One. 2016 Sep 16;11(9):e0162968. doi: 10.1371/journal.pone.0162968 (PMC5026350; doi:10.1371/journal.pone.0162968)
Supplement: S1 File — (ZIP) [file pone.0162968.s002.zip › S1_File/stubb/drosophila/output/gr_output/dmel_btd.fa.mfasta_1400.pdf]

# Chromosome dmel_btd.fa.mfasta, Position: 1400, Free en[illegible]

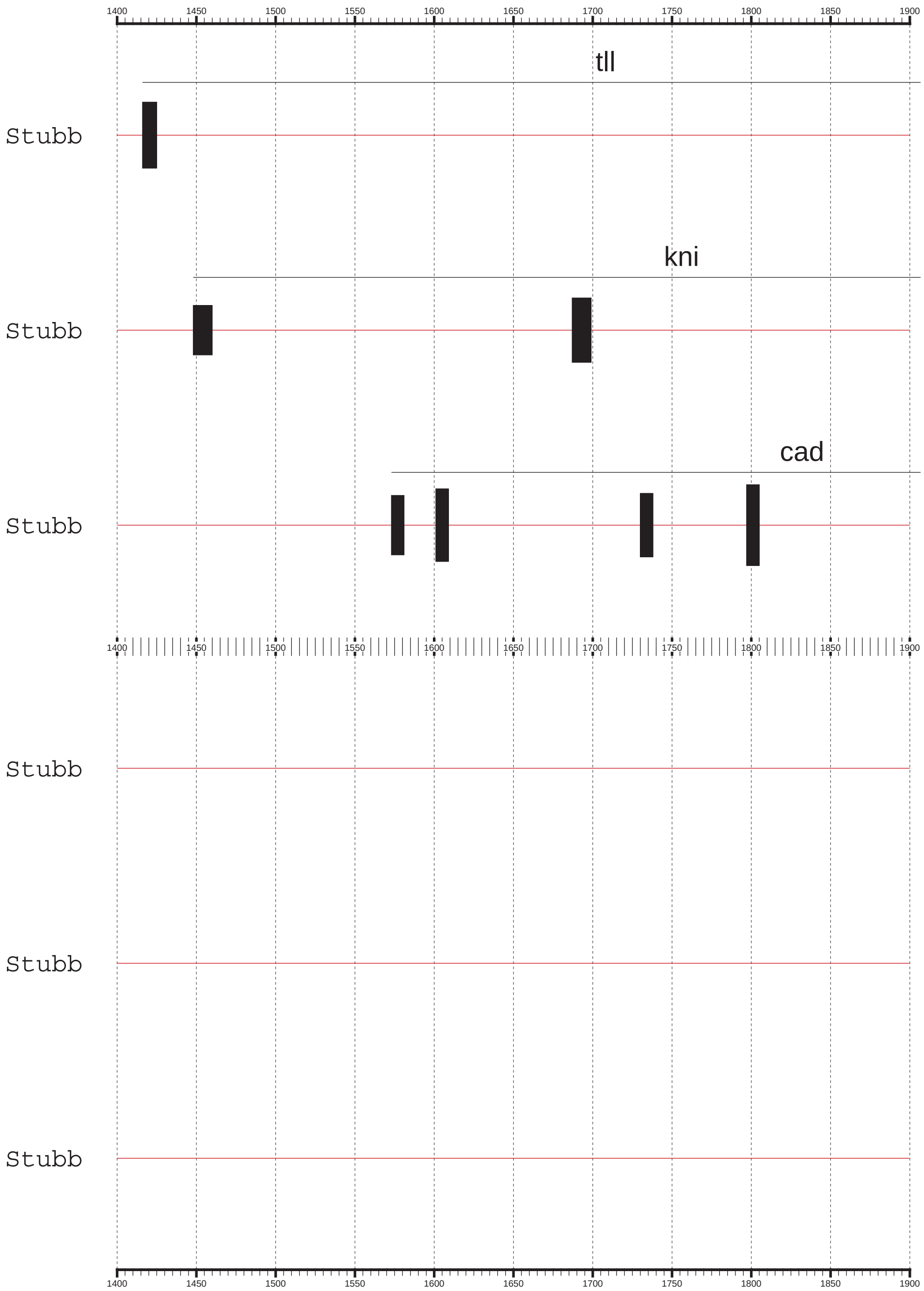


This plot has been obtained using gff2ps. The most recent version of gff2ps is freely available at "http://www1.imim.es/software/gfftools/GFF2PS.html". Copyright © 1999 by Josep F. Abril & Roderic Guigo
