# Supplementary material for: SMCis: An Effective Algorithm for Discovery of Cis-Regulatory Modules
Source: PLoS One. 2016 Sep 16;11(9):e0162968. doi: 10.1371/journal.pone.0162968 (PMC5026350; doi:10.1371/journal.pone.0162968)
Supplement: S1 File — (ZIP) [file pone.0162968.s002.zip › S1_File/stubb/liver/output/liver_peaks.html]

```
# Min_number_factors 3
# Free_energy_cutoff 5 Factor_min_occ 0.5
# Window_shift 50 Window_size 200
>
*********************** Rank 1 [Score 6.7437] PostScript Profile *******************
M19524	Position: 50	Nucl: 200	Word_av_length: 0.00	Free Energy: 6.7437
HNF-3          	0.0328	3.8257
HNF-4          	0.0243	2.8316
Background     	0.9378	109.4517
AF033857	Position: 600	Nucl: 200	
HNF-1          	0.0081	1.1457
HNF-3          	0.0301	4.2616
Background     	0.9618	136.2359
AF033857	Position: 650	Nucl: 200	
HNF-1          	0.0074	1.1528
HNF-3          	0.0191	2.9548
Background     	0.9735	150.8661
AF033857	Position: 700	Nucl: 200	
HNF-1          	0.0167	2.2648
HNF-3          	0.0216	2.9330
Background     	0.9590	129.9192
AF033857	Position: 750	Nucl: 200	
HNF-1          	0.0150	2.0011
HNF-3          	0.0237	3.1661
Background     	0.9558	127.6188
AF051355	Position: 500	Nucl: 200	
HNF-1          	0.0056	0.9708
HNF-4          	0.0060	1.0282
Background     	0.9884	170.6790
AF051355	Position: 550	Nucl: 200	
HNF-1          	0.0063	0.9891
HNF-4          	0.0076	1.1777
CEBP           	0.0075	1.1687
Background     	0.9786	152.5916
AF051355	Position: 600	Nucl: 200	
HNF-1          	0.0080	1.1485
CEBP           	0.0231	3.2947
Background     	0.9689	138.3903
AF051355	Position: 650	Nucl: 200	
HNF-1          	0.0090	1.0974
HNF-4          	0.0064	0.7814
CEBP           	0.0343	4.2014
Background     	0.9503	116.3767
AF236668	Position: 350	Nucl: 200	
HNF-4          	0.0132	2.2081
Background     	0.9854	164.4895
AF236668	Position: 400	Nucl: 200	
HNF-3          	0.0099	1.5229
HNF-4          	0.0153	2.3537
Background     	0.9747	149.4524
AF236668	Position: 450	Nucl: 200	
HNF-4          	0.0150	2.4214
Background     	0.9822	159.0601
AF236668	Position: 700	Nucl: 200	
HNF-1          	0.0148	1.9248
HNF-3          	0.0168	2.1889
CEBP           	0.0128	1.6674
Background     	0.9556	124.7573
AF236668	Position: 750	Nucl: 200	
HNF-1          	0.0153	1.8599
HNF-3          	0.0196	2.3847
CEBP           	0.0193	2.3483
Background     	0.9458	115.0703
AF236668	Position: 800	Nucl: 200	
HNF-1          	0.0105	1.5844
HNF-3          	0.0197	2.9592
Background     	0.9697	145.6833
L13460	Position: 450	Nucl: 200	
HNF-1          	0.0091	1.1845
HNF-3          	0.0323	4.1761
HNF-4          	0.0079	1.0210
Background     	0.9507	123.1108
L13460	Position: 500	Nucl: 200	
HNF-1          	0.0070	0.9869
HNF-3          	0.0224	3.1688
HNF-4          	0.0072	1.0123
Background     	0.9634	136.0330
L13460	Position: 550	Nucl: 200	
HNF-1          	0.0059	0.9851
HNF-3          	0.0130	2.1530
Background     	0.9811	162.6193
M15657	Position: 250	Nucl: 200	
HNF-1          	0.0144	1.8516
HNF-4          	0.0107	1.3753
CEBP           	0.0165	2.1295
Background     	0.9584	123.5745
M60197	Position: 300	Nucl: 200	
HNF-1          	0.0055	1.0265
Background     	0.9945	184.8501
M60197	Position: 350	Nucl: 200	
HNF-1          	0.0068	1.0612
HNF-3          	0.0100	1.5622
HNF-4          	0.0065	1.0229
Background     	0.9767	152.9518
M60197	Position: 400	Nucl: 200	
HNF-1          	0.0091	1.4070
HNF-3          	0.0100	1.5390
HNF-4          	0.0065	0.9964
Background     	0.9745	150.6255
M60197	Position: 450	Nucl: 200	
HNF-1          	0.0153	2.1629
HNF-3          	0.0081	1.1371
HNF-4          	0.0094	1.3285
Background     	0.9671	136.3486
U47685	Position: 750	Nucl: 200	
HNF-1          	0.0065	1.0225
HNF-3          	0.0178	2.8189
Background     	0.9757	154.3138
U47685	Position: 800	Nucl: 200	
HNF-1          	0.0065	1.0113
HNF-3          	0.0192	2.9988
Background     	0.9743	152.3019
<
```
