# Supplementary material for: SMCis: An Effective Algorithm for Discovery of Cis-Regulatory Modules
Source: PLoS One. 2016 Sep 16;11(9):e0162968. doi: 10.1371/journal.pone.0162968 (PMC5026350; doi:10.1371/journal.pone.0162968)
Supplement: S1 File — (ZIP) [file pone.0162968.s002.zip › S1_File/stubb/muscle/output/muscle_peaks.html]

```
# Min_number_factors 3
# Free_energy_cutoff 5 Factor_min_occ 0.5
# Window_shift 50 Window_size 200
>
*********************** Rank 1 [Score 25.8265] PostScript Profile *******************
M13631	Position: 50	Nucl: 200	Word_av_length: 0.00	Free Energy: 25.8265
sp1            	0.1333	9.0875
srf            	0.0587	3.9998
Background     	0.8081	55.0673
J04971	Position: 550	Nucl: 200	
myf            	0.0121	1.8204
sp1            	0.0120	1.8019
srf            	0.0066	0.9913
Background     	0.9694	146.1567
J04971	Position: 650	Nucl: 200	
mef2           	0.0065	1.0223
sp1            	0.0092	1.4496
srf            	0.0063	1.0044
Background     	0.9753	154.4553
J04971	Position: 700	Nucl: 200	
mef2           	0.0062	1.0082
sp1            	0.0058	0.9467
tef            	0.0073	1.1871
Background     	0.9780	158.6359
J04971	Position: 750	Nucl: 200	
mef2           	0.0059	1.0231
tef            	0.0078	1.3600
Background     	0.9863	171.8383
K01464	Position: 100	Nucl: 200	
mef2           	0.0055	1.0290
Background     	0.9945	187.7756
K01464	Position: 150	Nucl: 200	
mef2           	0.0055	1.0459
Background     	0.9943	187.4799
K01464	Position: 200	Nucl: 200	
mef2           	0.0057	1.0206
srf            	0.0054	0.9566
Background     	0.9888	176.1711
K01464	Position: 250	Nucl: 200	
mef2           	0.0060	1.0126
srf            	0.0093	1.5803
Background     	0.9843	167.1066
M20543	Position: 300	Nucl: 200	
sp1            	0.0342	4.8700
srf            	0.0071	1.0116
Background     	0.9587	136.4574
M20543	Position: 350	Nucl: 200	
sp1            	0.0645	6.9972
srf            	0.0208	2.2572
Background     	0.9147	98.9299
M20543	Position: 400	Nucl: 200	
sp1            	0.0699	7.1588
srf            	0.0228	2.3393
Background     	0.9073	92.8777
M20543	Position: 450	Nucl: 200	
sp1            	0.0333	3.7342
srf            	0.0307	3.4429
tef            	0.0092	1.0299
Background     	0.9268	103.7876
M20543	Position: 500	Nucl: 200	
mef2           	0.0106	0.9911
sp1            	0.0626	5.8664
srf            	0.0247	2.3110
tef            	0.0111	1.0449
Background     	0.8911	83.3925
M20543	Position: 550	Nucl: 200	
mef2           	0.0115	1.0048
myf            	0.0081	0.7125
sp1            	0.0841	7.3531
srf            	0.0129	1.1252
tef            	0.0114	0.9915
Background     	0.8720	76.0381
M20543	Position: 600	Nucl: 200	
mef2           	0.0129	1.0556
myf            	0.0282	2.3150
sp1            	0.0729	5.9985
srf            	0.0130	1.0699
tef            	0.0119	0.9764
Background     	0.8611	70.6085
M20543	Position: 650	Nucl: 200	
mef2           	0.0081	0.9742
myf            	0.0172	2.0674
sp1            	0.0407	4.8974
Background     	0.9339	112.0690
M21390	Position: 0	Nucl: 200	
mef2           	0.0057	0.9990
srf            	0.0059	1.0267
Background     	0.9866	171.9117
M21390	Position: 50	Nucl: 200	
mef2           	0.0070	1.0192
myf            	0.0071	1.0346
sp1            	0.0146	2.1191
srf            	0.0069	0.9990
Background     	0.9645	140.4061
M21390	Position: 100	Nucl: 200	
mef2           	0.0145	2.0185
myf            	0.0076	1.0519
sp1            	0.0113	1.5691
srf            	0.0072	1.0098
Background     	0.9594	133.6802
M21390	Position: 150	Nucl: 198	
mef2           	0.0143	2.0218
myf            	0.0081	1.1390
sp1            	0.0174	2.4581
Background     	0.9602	135.5176
M21390	Position: 200	Nucl: 198	
mef2           	0.0064	1.0068
myf            	0.0128	2.0234
sp1            	0.0047	0.7431
Background     	0.9761	154.0052
M22381	Position: 700	Nucl: 200	
myf            	0.0191	2.7104
sp1            	0.0148	2.0978
srf            	0.0055	0.7822
Background     	0.9606	136.5804
J04699	Position: 600	Nucl: 200	
myf            	0.0120	1.7469
sp1            	0.0253	3.6872
Background     	0.9627	140.4468
J04699	Position: 650	Nucl: 200	
myf            	0.0258	3.0434
sp1            	0.0334	3.9481
tef            	0.0101	1.1977
Background     	0.9307	109.9349
J04699	Position: 700	Nucl: 200	
myf            	0.0153	2.0351
sp1            	0.0265	3.5068
tef            	0.0087	1.1546
Background     	0.9494	125.9517
J04699	Position: 750	Nucl: 200	
myf            	0.0146	1.9757
sp1            	0.0239	3.2250
tef            	0.0079	1.0733
Background     	0.9536	129.1844
M57905	Position: 0	Nucl: 200	
mef2           	0.0067	1.0378
sp1            	0.0072	1.1207
srf            	0.0034	0.5319
tef            	0.0093	1.4502
Background     	0.9735	151.9223
M57905	Position: 50	Nucl: 200	
mef2           	0.0141	1.5180
sp1            	0.0374	4.0363
srf            	0.0136	1.4696
tef            	0.0186	2.0048
Background     	0.9164	98.9165
M57905	Position: 100	Nucl: 200	
sp1            	0.0496	5.9623
tef            	0.0169	2.0319
Background     	0.9335	112.1716
X14726	Position: 350	Nucl: 200	
mef2           	0.0211	2.8845
myf            	0.0218	2.9758
Background     	0.9571	130.7389
X14726	Position: 400	Nucl: 200	
mef2           	0.0230	3.0631
myf            	0.0240	3.1960
Background     	0.9529	126.7523
X14726	Position: 450	Nucl: 200	
mef2           	0.0248	3.2162
myf            	0.0166	2.1472
tef            	0.0087	1.1186
Background     	0.9499	123.1833
X14726	Position: 500	Nucl: 200	
mef2           	0.0218	3.1586
myf            	0.0072	1.0466
tef            	0.0058	0.8425
Background     	0.9652	140.2962
V01218	Position: 0	Nucl: 200	
srf            	0.0115	2.0159
Background     	0.9885	174.0417
V01218	Position: 50	Nucl: 200	
srf            	0.0118	2.0700
Background     	0.9881	173.7952
V01218	Position: 100	Nucl: 200	
srf            	0.0208	3.0775
tef            	0.0067	0.9866
Background     	0.9692	143.6824
V01218	Position: 150	Nucl: 200	
srf            	0.0137	2.1874
tef            	0.0091	1.4457
Background     	0.9764	155.4036
M63391	Position: 100	Nucl: 200	
mef2           	0.0064	0.9962
sp1            	0.0218	3.4112
Background     	0.9718	151.7635
M63391	Position: 150	Nucl: 200	
mef2           	0.0066	0.9977
myf            	0.0070	1.0578
sp1            	0.0172	2.6124
Background     	0.9693	147.2169
M63391	Position: 200	Nucl: 200	
mef2           	0.0062	1.0006
myf            	0.0072	1.1489
sp1            	0.0105	1.6741
Background     	0.9761	156.2806
M63391	Position: 250	Nucl: 200	
mef2           	0.0066	1.0081
myf            	0.0088	1.3464
sp1            	0.0153	2.3268
Background     	0.9693	147.7864
M63391	Position: 300	Nucl: 200	
myf            	0.0075	1.1723
sp1            	0.0208	3.2310
Background     	0.9716	150.8841
X12971	Position: 150	Nucl: 200	
myf            	0.0150	2.5812
Background     	0.9850	169.5715
X12971	Position: 250	Nucl: 200	
mef2           	0.0061	1.0492
myf            	0.0061	1.0409
tef            	0.0034	0.5850
Background     	0.9843	168.1665
X12971	Position: 550	Nucl: 200	
myf            	0.0111	1.5697
sp1            	0.0212	3.0085
srf            	0.0072	1.0254
Background     	0.9604	136.0063
X12971	Position: 600	Nucl: 200	
myf            	0.0148	1.9361
sp1            	0.0263	3.4359
srf            	0.0105	1.3768
Background     	0.9484	124.1947
X12971	Position: 650	Nucl: 200	
myf            	0.0120	1.8223
sp1            	0.0068	1.0314
srf            	0.0105	1.5863
Background     	0.9707	147.3236
X12971	Position: 700	Nucl: 200	
myf            	0.0079	1.2059
sp1            	0.0064	0.9830
srf            	0.0134	2.0586
Background     	0.9722	148.8885
X12971	Position: 800	Nucl: 200	
myf            	0.0126	1.9368
srf            	0.0153	2.3512
Background     	0.9720	149.1602
M95800	Position: 250	Nucl: 199	
sp1            	0.0240	3.8632
Background     	0.9759	156.9683
M95800	Position: 350	Nucl: 200	
mef2           	0.0062	1.0316
myf            	0.0060	1.0060
sp1            	0.0064	1.0772
Background     	0.9814	164.4667
M95800	Position: 400	Nucl: 200	
mef2           	0.0063	1.0358
myf            	0.0135	2.2286
Background     	0.9802	161.5694
M95800	Position: 450	Nucl: 200	
mef2           	0.0064	1.0655
myf            	0.0124	2.0669
Background     	0.9811	162.8910
M95800	Position: 500	Nucl: 200	
mef2           	0.0063	1.0974
myf            	0.0060	1.0598
Background     	0.9870	173.0876
L21905	Position: 350	Nucl: 200	
mef2           	0.0169	2.6268
myf            	0.0094	1.4574
Background     	0.9737	151.2815
L21905	Position: 400	Nucl: 200	
mef2           	0.0164	2.6437
myf            	0.0061	0.9871
Background     	0.9774	157.1713
L21905	Position: 450	Nucl: 200	
mef2           	0.0117	1.9767
myf            	0.0056	0.9507
Background     	0.9826	165.3798
U02285	Position: 0	Nucl: 200	
sp1            	0.0391	5.5111
Background     	0.9578	134.8631
U02285	Position: 50	Nucl: 200	
sp1            	0.0706	8.4480
Background     	0.9294	111.1431
U02285	Position: 100	Nucl: 200	
sp1            	0.0445	6.1792
Background     	0.9555	132.7720
U02285	Position: 250	Nucl: 200	
myf            	0.0052	0.7502
sp1            	0.0294	4.2359
tef            	0.0057	0.8280
Background     	0.9596	138.5020
U02285	Position: 300	Nucl: 200	
myf            	0.0063	0.8406
sp1            	0.0364	4.8795
tef            	0.0056	0.7502
Background     	0.9517	127.8157
U02285	Position: 350	Nucl: 200	
myf            	0.0054	0.6369
sp1            	0.0606	7.1440
tef            	0.0070	0.8315
Background     	0.9270	109.1920
U02285	Position: 400	Nucl: 200	
sp1            	0.0349	4.8313
srf            	0.0071	0.9792
Background     	0.9560	132.3493
U02285	Position: 450	Nucl: 200	
sp1            	0.0352	4.5945
srf            	0.0160	2.0940
Background     	0.9488	124.0812
U02285	Position: 500	Nucl: 200	
sp1            	0.0341	4.1839
srf            	0.0261	3.2068
Background     	0.9398	115.3846
U02285	Position: 550	Nucl: 200	
sp1            	0.0340	4.1514
srf            	0.0256	3.1271
Background     	0.9404	114.9368
U02285	Position: 600	Nucl: 200	
mef2           	0.0098	0.9916
sp1            	0.0654	6.6140
srf            	0.0223	2.2489
Background     	0.9025	91.0394
U02285	Position: 650	Nucl: 200	
mef2           	0.0071	0.9977
sp1            	0.0275	3.8460
srf            	0.0071	0.9916
Background     	0.9583	134.1605
U02285	Position: 700	Nucl: 200	
mef2           	0.0068	0.9655
sp1            	0.0333	4.7368
Background     	0.9599	136.6210
U02285	Position: 750	Nucl: 200	
sp1            	0.0229	3.7642
Background     	0.9771	160.6045
U02285	Position: 800	Nucl: 200	
sp1            	0.0262	4.1809
Background     	0.9738	155.6382
M62404	Position: 100	Nucl: 200	
mef2           	0.0056	1.0562
Background     	0.9944	187.5424
M62404	Position: 150	Nucl: 200	
mef2           	0.0056	1.0433
Background     	0.9939	186.6823
M62404	Position: 200	Nucl: 200	
mef2           	0.0101	1.7079
tef            	0.0059	0.9993
Background     	0.9828	165.8777
M62404	Position: 250	Nucl: 200	
mef2           	0.0113	1.6747
sp1            	0.0128	1.8921
tef            	0.0069	1.0249
Background     	0.9660	142.7394
X62155	Position: 100	Nucl: 200	
mef2           	0.0068	1.0037
myf            	0.0060	0.8880
sp1            	0.0224	3.3104
Background     	0.9649	142.8218
X62155	Position: 150	Nucl: 200	
mef2           	0.0075	1.1078
myf            	0.0155	2.2966
sp1            	0.0105	1.5578
Background     	0.9666	143.4767
X62155	Position: 200	Nucl: 200	
mef2           	0.0063	1.0363
myf            	0.0139	2.2800
Background     	0.9794	160.2596
X62155	Position: 250	Nucl: 200	
mef2           	0.0060	1.0366
myf            	0.0068	1.1766
Background     	0.9846	169.2123
X62155	Position: 700	Nucl: 200	
myf            	0.0088	1.3283
sp1            	0.0222	3.3734
Background     	0.9690	147.3078
X62155	Position: 750	Nucl: 199	
sp1            	0.0268	4.2197
Background     	0.9732	153.2326
X62155	Position: 800	Nucl: 199	
sp1            	0.0272	4.2784
Background     	0.9727	152.8270
M13483	Position: 100	Nucl: 200	
sp1            	0.0486	5.6332
srf            	0.0217	2.5132
Background     	0.9297	107.8287
M13483	Position: 150	Nucl: 200	
sp1            	0.0596	5.8916
srf            	0.0377	3.7260
Background     	0.9027	89.2160
M13483	Position: 200	Nucl: 200	
sp1            	0.0443	4.7189
srf            	0.0397	4.2273
Background     	0.9159	97.4539
M13483	Position: 250	Nucl: 200	
sp1            	0.0292	3.7204
srf            	0.0243	3.1034
Background     	0.9464	120.6366
M13483	Position: 300	Nucl: 200	
sp1            	0.0159	2.4016
srf            	0.0152	2.2949
Background     	0.9689	146.2035
M13483	Position: 350	Nucl: 200	
sp1            	0.0333	4.6645
srf            	0.0083	1.1651
Background     	0.9584	134.3769
X73887	Position: 250	Nucl: 200	
myf            	0.0187	2.5856
sp1            	0.0106	1.4676
srf            	0.0044	0.6059
tef            	0.0080	1.1079
Background     	0.9582	132.8049
X73887	Position: 300	Nucl: 200	
myf            	0.0218	2.7165
sp1            	0.0227	2.8200
srf            	0.0049	0.6066
tef            	0.0091	1.1383
Background     	0.9411	117.2319
X67686	Position: 550	Nucl: 200	
sp1            	0.0065	1.0736
srf            	0.0123	2.0258
Background     	0.9813	162.2110
X67686	Position: 600	Nucl: 200	
sp1            	0.0074	1.1198
srf            	0.0215	3.2408
Background     	0.9710	146.1773
X67686	Position: 650	Nucl: 200	
sp1            	0.0204	2.4453
srf            	0.0355	4.2635
tef            	0.0072	0.8590
Background     	0.9370	112.5761
X67686	Position: 700	Nucl: 200	
sp1            	0.0206	2.6731
srf            	0.0194	2.5145
tef            	0.0082	1.0607
Background     	0.9496	123.2832
<
```
